# Omp34-Mediated *Acinetobacter baumannii* Invasion of Human Cervical Carcinoma Epithelial, HeLa Cells, and the Influence of Anti-Omp34 Antibodies

**DOI:** 10.1155/ancp/1931119

**Published:** 2025-04-10

**Authors:** Seyedeh Faezeh Hosseini, Mohammadreza Jalali Nadoushan, Zahra Fekrirad, Iraj Rasooli

**Affiliations:** ^1^Department of Biology, Shahed University, Tehran, Iran; ^2^Department of Pathology, School of Medicine, Shahed University, Tehran, Iran; ^3^Molecular Microbiology Research Center and Department of Biology, Shahed University, Tehran, Iran

**Keywords:** *Acinetobacter baumannii*, adherence, HeLa, internalization, Omp34

## Abstract

*Acinetobacter baumannii* is known for its ability to invade and persist within eukaryotic cells, impacting infection outcomes and disease progression. This study investigates the role of Omp34, a key outer membrane protein (Omp), in *A. baumannii* interaction with epithelial cells and the protective effects of anti-Omp34 antibodies (Abs). Omp34 is a key regulator of *A. baumannii* epithelial cell invasion, influencing bacterial adherence, internalization, and intracellular proliferation. The presence of anti-Omp34 Abs mitigates *A. baumannii*-induced cellular damage and enhances bacterial clearance. The process involved the expression and purification of Omp34, which in turn induced Abs in BALB/c mice against Omp34. The acute toxicity of Omp34 was studied through a histological analysis conducted on six distinct organs in mice. HeLa cells were infected by *A. baumannii* ATCC 19606 and a clinical strain. Various aspects of *A. baumannii* behavior with HeLa cells, including HeLa cell viability, adherence, serum resistance, cell internalization, and intracellular proliferation with and without anti-Omp34 sera. Cytoskeleton inhibitors were used to study the potential roles played in the process of *A. baumannii* invasion by microfilaments and microtubules. Omp34 effectively triggered Ab production in mice without resulting in any toxicity. The assay for serum resistance revealed potent bactericidal and antibiofilm effects on both *A. baumannii* strains. Bacterial internalization was constrained when actin polymerization was inhibited. Examination under the microscope revealed instances of adherence, alterations in the cell membrane, apoptosis, vacuolization, and cell damage. HeLa cells exposed to anti-Omp34 serum showed decreased cell damage. The results provide substantial evidence of the adherence capacity of *A. baumannii* to proliferate in the epithelial cells. In conclusion, Omp34 plays a substantial role in regulating interactions between epithelial cells and *A. baumannii*, the multifaceted nature of which intricately modifies the trajectory of infection within host cells by *A. baumannii*.

## 1. Introduction

*Acinetobacter baumannii* is a gram-negative bacillus commonly found in aerobic environments [1]. Due to its extensive drug resistance, it is categorized as an ESKAPE pathogen, posing a substantial threat to global public health [2]. This versatile pathogen can thrive in diverse environments and can form biofilms on both biotic and abiotic surfaces. *A. baumannii* is commonly associated with hospital-acquired infections, particularly those associated with ventilators or catheters. Clinical manifestations resulting from *A. baumannii* infections vary from bacteremia to pneumonia, urinary tract infections (UTIs), soft tissue infections, and intra-abdominal infections [3]. *A. baumannii* deploys various mechanisms to counteract the effects of antibiotics, including the production of β-lactamases, aminoglycoside-modifying enzymes, target site modification, efflux pumps, and permeability defects. Although the overall occurrence of *A. baumannii* infections might be comparatively low when compared to other gram-negative bacteria, the prevalence of multidrug-resistant strains in *A. baumannii* is notably higher, surpassing that of bacteria like *Klebsiella pneumoniae* and *Pseudomonas aeruginosa* by four times [4]. These factors highlight the urgent need to address *A. baumannii* infections and occurrence of antibiotic resistance to mitigate the detrimental effects on both human health and the economy. Strategies focusing on infection prevention, appropriate antibiotic stewardship, and finding new treatment options are essential to combat the clinical and economic consequences of *A. baumannii* infections [5]. Vaccination represents an effective role in preventing and controlling *A. baumannii* infections [6]. Recently, extensive research was conducted to uncover the intricate mechanisms underlying *A. baumannii* emergence in clinical settings. Unlike other nosocomial pathogens like *P. aeruginosa*, *E. coli*, *S. aureus*, and *A. baumannii* follow a distinct pathogenic strategy. The capacity to induce disease is attributed to a combination of factors and the coordinated action of various virulence factors. The initial stage of infection involves the interaction between the surface-exposed molecular components on *A. baumannii* cells and the surrounding environment. The surface of *A. baumannii* contains a diverse range of molecular constituents that facilitate these interactions [7]. Despite the considerable threat that *A. baumannii* poses to public health, the vaccine development for this pathogen is trailing behind efforts targeting other pathogens.

Its recognition as a major public health threat has driven efforts to develop new therapeutic strategies and infection control measures. Currently, available antimicrobial agents are becoming increasingly ineffective as bacteria continually evolve new resistance mechanisms. The battle against *A. baumannii* extends beyond microbiological research and hospital settings. It is a pressing global public health challenge that requires a coordinated and comprehensive response [8]. Outer membrane of gram-negative bacteria is a selective barrier, primarily owing to the presence of specialized membrane-spanning proteins referred to as “porins.” Porins adopt a β-barrel shape, forming channels that facilitate the passive movement of hydrophilic molecules, while effectively impeding the passage of large and charged molecules. Typically, these porins organize themselves within the outer membrane in sets of three. They are highly abundant on the bacterial surface and thus have diverse and significant roles in interactions between the host and bacteria. Some of these roles encompass facilitating adhesion and virulence mechanisms that contribute to the development of infections. Porins also enhance the bacteria's resistance against antimicrobial agents. Moreover, they play as receptors for complement proteins and phages and play a part in regulating the host cell responses. Furthermore, researchers are now exploring the potential of porin applications as vaccine candidates for therapeutic interventions, adjuvants, and biomarkers.

The collection of virulence factors in *A. baumannii*, which includes components of outer membrane proteins (Omps), cellular envelope, phospholipases, secretion systems, and the ability to form biofilms, empowers the bacterium to endure challenging environmental circumstances over prolonged durations. This capability improves its ability to establish itself and cause infections in vulnerable hosts [7]. A significant virulence factor of *A. baumannii* is its Omp, specifically Omp33–36, also identified as Omp33 or Omp34. This protein features a 14-strand beta-barrel structure and is involved in host cell adhesion, as well as the initiation of host cell apoptosis through the activation of caspases [9–11]. Omp34 demonstrates a high level of conservation among *A. baumannii* strains, exhibiting a remarkable 98% similarity across more than 1600 strains [11]. In experiments involving Omp34 immunization in BALB/c mice models, a notable rise in IgG titers and a decrease in bacterial burden were noted, resulting in the complete elimination of bacteria from the spleen and liver of mice [12]. Omp33−36, along with OmpA and TonB, are collectively referred to as fibronectin-binding proteins (FBPs) [13] associated with the adhesion, invasion, cytotoxicity, and metabolic adaptability of *A. baumannii* [10]. IgM, IgA, and IgG from patients infected with *A. baumannii* specifically recognize Omp33−36, and there is no cross-reactivity observed with sera from patients infected with pathogens other than *A. baumannii* [14]. The sequences of Omp33−36 demonstrated a ≥98% identity among over 1670 strains of *A. baumannii* [11]. As a highly conserved, nontoxic, and immunogenic protein in *A. baumannii*, Omp33−36 provides partial to complete protection against infection caused by *A. baumannii*. While it is currently under preclinical study, its potential for clinical transformation is evident. It is important to acknowledge that Omp33−36 may not provide complete protection in all challenge models, and there is promise in exploring novel adjuvants, including nanomaterials and bacterial outer membrane vesicles, to enhance its immunogenicity [15]. Recently discovered porins in *A. baumannii* include Omp34, previously known as Omp33−36. Similar to OmpA, Omp34 plays a role in bacterial pathogenesis by adhering to and inducing death in lung epithelial cells through caspase activation [9, 10]. A proposed virulence protein, Omp33−36, has been suggested to induce incomplete autophagy, thereby facilitating the replication of *A. baumannii* [9]. Over the past decades, substantial progress has been made in comprehending the pathogenesis of *A. baumannii*, encompassing its virulence factors, mechanisms of antimicrobial resistance, and quorum sensing [16, 17]. The porin Omp33−36 emerged as the virulence factor responsible for triggering apoptosis through the activation of caspases, as well as inducing autophagy, as evidenced by the buildup of p62 and LC3B-II [9]. Consequently, there exists a pressing demand for the exploration and creation of innovative or unconventional methods suitable for addressing infections linked with *A. baumannii*. Therapeutic strategies involving the elimination or eradication of microorganism populations by introducing antagonistic microorganisms or their secondary metabolites are gaining greater attention as potential solutions [18]. Pathogenic bacteria that circulate within the bloodstream must establish a mechanism to engage with the endothelial cells (ECs) that form the lining of blood vessels. This interaction is crucial for these bacteria to initiate infection and establish colonization within the host. For deep understanding of the pathogenesis and virulence of *A. baumannii*, as well as its interaction with the host during infection, it is essential to utilize host–pathogen interaction models. Animal models have been extensively employed in studying *A. baumannii* pathogenesis, given diverse clinical manifestations. This paper provides a concise exploration of porin protein Omp34, its vital function, and involvement in interactions between hosts and bacteria.

## 2. Materials and Methods

### 2.1. Bacteria and Human Cervical Carcinoma Epithelial (HeLa) Cells

*A. baumannii* ATCC 19606 and its clinical isolated strain, *A. baumannii* 58ST, *E. coli* BL21 (DE3) harboring pET-28 with *omp*34 gene corresponding to 34 kDa of Omp34 (accession number WP_000733005.1), were grown at 37°C in Luria–Bertani (LB) broth. The human cervical carcinoma epithelial cells, HeLa (ATCC CCL-2), was procured from the National Cell Bank of Iran, Pasteur Institute of Iran (Tehran), and cultured as described by An et al. [19]. Omp34 clone was from our previous work, and the protein expression and purification protocols were performed following the same methodology [12].

### 2.2. Mice Groups

Male BALB/c mice within 20–25 g weight range were sourced from the Royan Institute, Tehran. These animals were grouped as control and the Omp34. These major groups were further subdivided into two subgroups for immunization and for acute cytotoxicity assessment. Each subgroup contained five mice. The experimental animals were maintained in a controlled environment in the specific pathogen-free (SPF) animal house of Shahed University with a 12:12-h light/dark cycle, a temperature range of 22–23°C, and 40% humidity level. They were provided a standard antibiotic-free diet and unlimited access to water.

### 2.3. Immunization of Mice

The mice were administered subcutaneously with purified protein on days 0, 14, 28, and 42. The initial injection included a concentration of 20 μg/mL of protein along with complete adjuvant. Subsequent injections utilized incomplete adjuvant. Blood samples were collected before each immunization and processed to obtain blood sera to use in indirect enzyme-linked immunosorbent assay (ELISA) test as described earlier [12].

### 2.4. Omp34 Toxicity

Acute toxicity of Omp34 was assessed using BALB/c mice [20]. Mice that were 8 weeks old (*n* = 4/group) received either phosphate-buffered saline (PBS) (control group) or Omp34 at 20, 50, or 500 μg doses per mouse via subcutaneous route. The mice groups were monitored for any alterations in clinical indicators. Blood and tissue samples including the brain, liver, spleen, heart, lung, and kidney were processed as described earlier [21].

### 2.5. Anti-Omp34 Antibodies (Abs) and *A. baumannii* Resistance

Serum resistance assays were conducted following previously established procedures [22], with minor adjustments as outlined in our previous work [21]. The percent survival is presented by calculating the findings in triplicate to express the serum resistance as a viability ratio. The standard deviations (SDs) are depicted as error bars. In the context of statistical significance, “*⁣*^*∗*^,” is used to indicate a significant difference (*p*  < 0.05).

### 2.6. Inhibition of Biofilm Formation

The effect of monoclonal Abs on the formation of biofilm was assessed, following a methodology outlined by She et al. [23] with certain modifications. In brief, 100 μL of a pool of Abs diluted to 1:250, 1:500, and 1:1000 in tryptic soy broth (TSB) was coated in each well of a 96-well polystyrene plate. Ten microliter of 10^8^ colony-forming unit (CFU)/mL of bacterial strains prepared in 0.9% NaCl solution was added to each well. Wells lacking serum samples were employed as positive controls for bacterial growth, while wells containing only saline solution were used as negative controls and incubated for 18–24 h at 37°C. One hundred twenty microliter of methanol P.A. (99.9%) was added for 20 min to fix the formed biofilm. After the complete drying of the biofilm, 0.5% crystal violet solution was added followed by loosening the biofilm with the addition of 120 μL of 95% ethanol and allowing to stand for 30 min. Absorbance at OD_570_ was then recorded.

### 2.7. *A. baumannii* Strain Adherence, Internalization, and Proliferation

Previously established methods were used for conducting adherence and invasion experiments [24, 25]. In brief, the HeLa cell line was cultured on plastic coverslips of 13-mm diameter in 24-well plates. The cells were grown at 37°C for 16–24 h in Dulbecco's Modified Eagle's Medium (DMEM) supplemented with 2 mM L-glutamine and 10% fetal calf serum. *A. baumannii* strains grown in LB broth were separately washed in PBS and adjusted to ∼5 × 10^9^ CFU/mL at OD_620_ = 0.5. These suspensions were used to infect the HeLa cells for ∼100% cell confluence in triplicate at a multiplicity of infection (MOI) of ~100:1. The monolayers were then incubated for 22 h at 37°C in a 5% CO_2_ followed by washing thrice with sterile prewarmed PBS and were immediately fixed with 50% ethanol and processed for Papanicolaou staining. The coverslips were mounted on grease-free clean glass slide cells and examined under Zeiss compound microscope at 100× magnification. Bacterial adherence was assessed through the examination of about 500 HeLa cells per coverslip cell under the light microscope. The percent epithelial cells adhered by at least a single bacterium was calculated. To evaluate bacterial adherence to HeLa cells, the total bacteria adhered to 100 cells were enumerated. HeLa cells infected with *Salmonella enterica* serovar Enteritidis strain at an MOI of ~100:1 for 90 min served as positive control. To ascertain whether *Acinetobacter* strains exhibit adherence preference for cell or inert surfaces, HeLa cells were seeded at a confluency of 100%. By assessing the CFUs on coverslips with varying cell densities (exposing inert surfaces), we could discern *Acinetobacter*'s inclination toward either the cellular or inert surface. Elevated CFUs on coverslips with more cells/less inert surface exposed (~100% confluence) would imply a preference for the cellular surface, while increased CFUs on coverslips with fewer cells/more inert surface exposed (~50% confluence) would suggest a preference for the inert surface. HeLa cells were seeded in 24-well tissue culture plates at around 100% confluence to conduct survival of both *A. baumannii* strains within the host cell. HeLa cells were then incubated for 4 h. One hundred μg/mL of gentamicin was added for 2 h to eliminate extracellular bacteria followed by washing and replacing with fresh medium containing gentamicin (10 μg/mL). The cells were incubated for an additional 20 h prior to lysis. Viable intracellular bacteria were then quantified. Adherent bacteria were determined by removing nonadherent external bacteria through four PBS washes. Cell disruption was achieved by adding 100 μL of a 1% Triton X-100 in PBS solution per coverslip or well. The resulting disrupted mixture underwent serial dilution and was plated on LB agar, followed by incubation at 37°C for 24 h. Bacterial adherence was assessed as the precent CFUs per the initial total inoculum. To ensure robustness and account for experimental variability, these adherence experiments were repeated three times.

The intracellular proliferation rate (Ipro) is defined as the viable intracellular bacteria ratio at 24 h by 4 h postinfection [26, 27]. The percent efficiency of invasion was assessed by calculating the average CFUs per initial total inoculum. The monolayers infected with *A. baumannii* were monitored under a light microscopy for 24 h. *E. coli* DH5-α and *S. enterica* serovar typhimurium served as a noninvasive negative and positive controls, respectively. All experiments were conducted in triplicate. To ensure statistical validity and account for potential variations, the entire set of experiments was repeated three times.

### 2.8. Impact of Sera From Immunized Mice on Bacterial Adherence to HeLa Cells

HeLa cells at 1 × 10^5^ cells/well seeded in 24-well plates were exposed to each of the bacterial strains pre-exposed to anti-Omp34 serum at 1:250 dilution following the methods described in the “Anti-Omp34 antibodies and resistance of *A. baumannii* strains” [28]. The serum-exposed cells were incubated for 24 h for interactions between bacteria and cells. For quantification of cell viability, the optical density (OD) measurements were placed in (absorbance of treated cells/mean absorbance of nontreated cells) × 100.

### 2.9. HeLa Cell Infection With Killed *A. baumannii*

Fresh bacterial cells grown over night in LB broth on rotary shaker at 37°C were centrifuged at 3000 g/30 min, washed thrice with PBS (pH 7.2), adjusted to ~5 × 10^9^ CFU/mL in PBS, and were equally divided into four batches. Three killing methods were employed. Four hundred microliter of each bacterial suspension was exposed to 80°C for 25 min in water bath. A 5 mL suspension was exposed to UV light for a duration of 2 h. Formalin was added to the bacterial suspension to a final concentration of 0.35% and incubated shaking at 4°C for 16 h followed by PBS wash and readjustment to ~5 × 10^9^ CFU/mL in PBS. To confirm bacterial death, LB agar plates were streaked with the killed bacterial suspension in triplicate. Once bacterial death was confirmed, 10 μL from each aliquot was withdrawn to infect our sets of HeLa cells in triplicate at the MOI of about 100%. The cells were stained with Papanicolaou stain, observed at 100× magnification.

### 2.10. Bacterial Infection and HeLa Cell Viability

Cellular viability of the infected HeLa cells was assessed quantitatively by monitoring mitochondrial reduction activity using the 3-(4,5-dimethylthiazol-2-yl)-2,5-diphenyltetrazolium bromide (MTT) assay, as previously described [29].

### 2.11. Effect of Actin Disruption on Invasion of HeLa Cells by *A. baumannii*

The cellular invasion was confirmed by 1 μg/mL of an actin-disrupting agent, cytochalasin D. Before gentamicin treatment, the HeLa cells were preincubated with cytochalasin D for 30 min at 37°C. This preceded infection and was maintained throughout the duration of infection. Cytochalasin D inhibits bacterial uptake by the cells. Following cytochalasin D preincubation, two approaches were employed. In the first approach, cells were extensively washed to remove noninvaded bacteria. In the second approach, cells were maintained at a temperature of 4°C to halt further bacterial internalization. The percent internalized bacterial population was quantified by dividing the total count of internalized bacteria within cytochalasin D treated HeLa cells by the number of internalized bacteria within untreated HeLa cells [28].

### 2.12. Statistical Analysis and Data Presentation

Statistical analyses and graph creation were performed using GraphPad Prism 9.0.0. The data presentation was mean values accompanied by their respective SDs. Shapiro-Wilkinson test was used to assess the normality of all datasets. One-way analysis of variance (ANOVA) test with Holm–Sidak's correction was utilized for multiple comparisons upon confirmation of normal distribution. Two-way ANOVA test was employed for scenarios involving two independent variables. Kruskal–Wallis test was applied, followed by Dunn's correction where data did not exhibit normality. Significance was determined by comparing calculated *p*-values to a threshold of 0.05. The significance level was denoted by asterisks: *⁣*^*∗*^*p*  < 0.05, *⁣*^*∗∗*^*p*  < 0.01, *⁣*^*∗∗∗*^*p* < 0.001, and *⁣*^*∗∗∗∗*^*p*  < 0.0001.

## 3. Results

### 3.1. Toxicity in Immunized Mice

**Figure 1:**
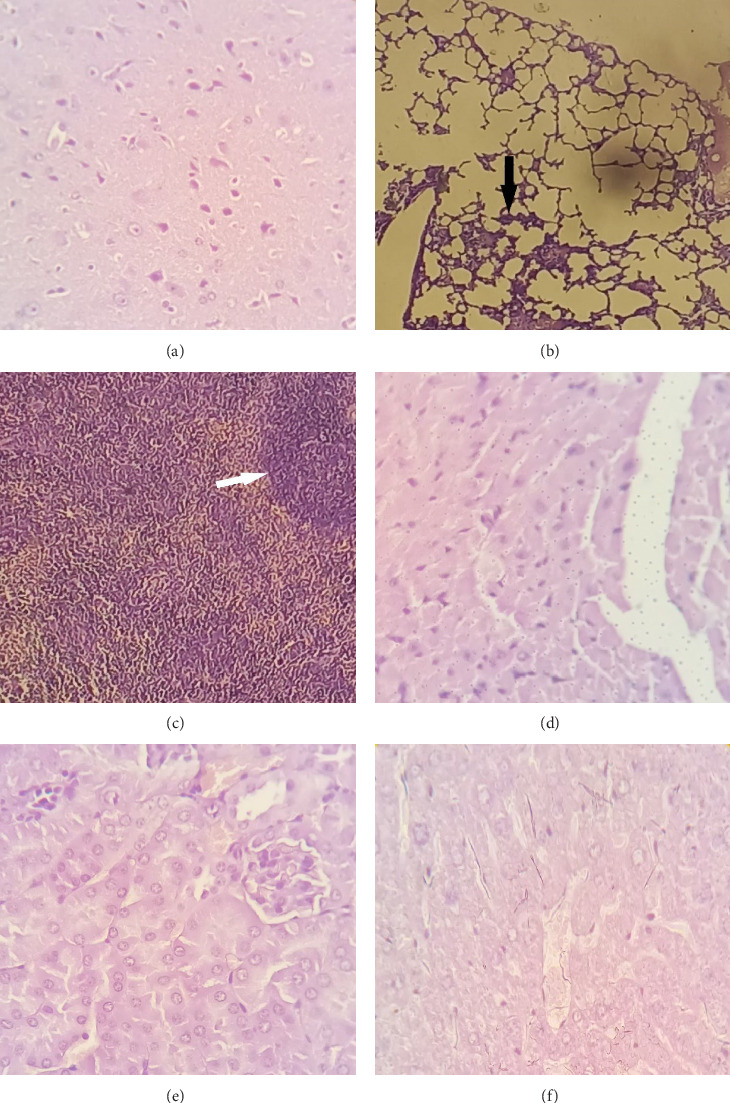
Histological analysis of mice internal organs. (A) Normal brain tissue (×400). (B) Mild mononuclear leukocytic infiltration in alveolar walls of the lungs (×400), indicated by an arrow. (C) Mild nonspecific inflammation in the spleen (×100), marked by an arrow. (D) Normal heart structure. (E) Kidney sections showing normal histology. (F) Liver tissue exhibiting a normal structure.

The tissue sections were stained with hematoxylin and eosin (H&E) for histopathological assessment as outlined previously [21]. The results are depicted in Figure 1.

### 3.2. Ab Response and Serum Resistance Assay

**Figure 2:**
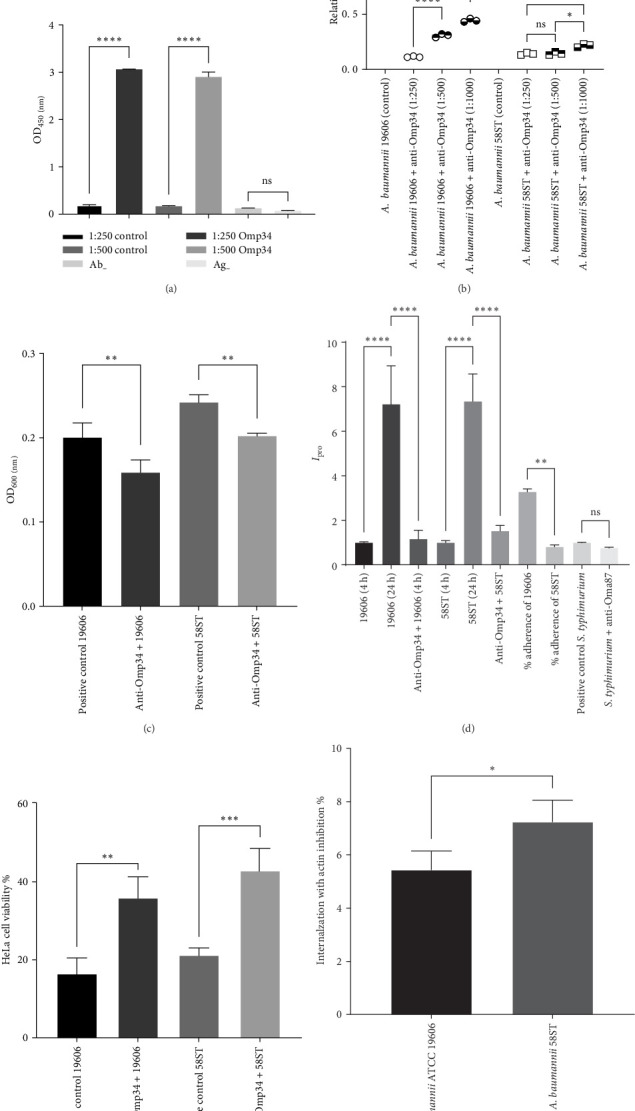
(A) Indirect ELISA of mice antisera raised against Omp34. Wells were coated with 5 µg of recombinant Omp34, and absorbances (OD_450_) were measured at 1:250 and 1:500 dilutions using immunized mice sera. Data represent the mean of three independent experiments performed in triplicate, with standard error of the mean (SEM) indicated. While both dilutions exhibited elevated levels of specific antibodies, no significant difference was observed between them. However, antibody levels were significantly higher compared to the control group. These differences are shown as *⁣*^*∗*^*p*  < 0.05, *⁣*^*∗∗*^*p*  < 0.01, *⁣*^*∗∗∗*^*p*  < 0.001, and *⁣*^*∗∗∗∗*^*p*  < 0.0001. ns stands for “not significant.” The lower and upper confidence intervals (CIs) are 1:250 control (0.1796–0.2094), 1:250 Omp34 (3.058–3.072), 1:500 control (0.1473–0.1928), 1:500 Omp34 (2.763–3.044), Ab^−^ (0.1157–0.1407), and Ag^−^ (0.06362–0.09049). (B) Serum resistance of *A. baumannii* strains. Serum resistance is represented as the viability ratio (colony-forming units [CFUs] of the serum bacterial suspension to CFUs of the bacterial suspension without serum). The figure illustrates the relative quantity of *A. baumannii* treated with anti-Omp34 serum. Error bars represent standard deviations. Statistically significant differences are indicated as *p* < 0.05, *p* < 0.01, *⁣*^*∗*^*p* < 0.001, and *⁣*^*∗∗*^*p* < 0.0001. The lower and upper CIs are *A. baumannii* 19606 control (0.9104–1.090), *A. baumannii* 19606 + anti-Omp34 of 1:250 dilution (0.099–0.1277), *A. baumannii* 19606 + anti-Omp34 of 1:500 dilution (0.2687–0.3446), *A. baumannii* 19606 + anti-Omp34 of 1:1000 dilution (0.4054–0.4813), *A. baumannii* 58ST control (0.9006–1.099), *A. baumannii* 58ST + anti-Omp34 of 1:250 dilution (0.1152–0.1648), *A. baumannii* 58ST + anti-Omp34 of 1:500 dilution (0.1054–0.1813), *A. baumannii* 58ST + anti-Omp34 of 1:1000 dilution (0.1787–0.2546). (C) Effect of anti-Omp34 serum on biofilm formation ability of *A. baumannii* strains. Positive control 19606: *A. baumannii* ATCC 19606 without serum. The lower and upper CIs are 0.1580–0.2429. Anti-Omp34 + 19606: *A. baumannii* ATCC 19606 mixed with anti-Omp34 serum (1:250). CI = 0.1220–1958. Positive control 58ST: *A. baumannii* 58ST without serum. CI = 0.21904–0.2647. Anti-Omp34 + 58ST: *A. baumannii* 58ST mixed with anti-Omp34 serum (1:250). CI = 0.1940–0.2104. Statistically significant differences are indicated as *p* < 0.05, *p* < 0.01, *⁣*^*∗*^*p* < 0.001, and *⁣*^*∗∗*^*p* < 0.0001. (D) Bacterial attachment, uptake, and growth within host cells. Cells were infected for 4 h, and the intracellular proliferation rate (*I*_pro_) was determined by measuring the ratio of viable intracellular bacteria at 24 h to those at 4 h postinfection. Each assay was performed in triplicate, and the results are expressed as the mean ± SE from three independent experiments. To allow for comparative visualization in the graph, bacterial adherence percentages were plotted at 10% of their original values. As illustrated, anti-Omp34 affected the proliferation of both strains of bacteria significantly (*p*  < 0.0001). The bacterial strains had 32.87 ± 1.21% (*A. baumannii* 19606) and 8.17 ± 0.76% (*A. baumannii* 58ST) adherence. The positive control that is *S. enterica* serovar typhimurium also showed a nonsignificant reduction in intracellular proliferation. The Holm–Šídák test was used for comparisons, with significant differences indicated as *⁣*^*∗*^*p*  < 0.05, *⁣*^*∗∗*^*p*  < 0.01, *⁣*^*∗∗∗*^*p*  < 0.001, and *⁣*^*∗∗∗∗*^*p*  < 0.0001. The lower and upper CIs are 19606 (4 h) = (0.9094–1.084), 19606 (24 h) (2.942–11.50), anti-Omp34 + 19606 (0.2157–2.118), 58ST (4 h) (0.7723–1.228), 58ST (24 h) (4.304–10.39), anti-Omp34 + 58ST (0.9211–2.132), % adherence of 19606 (2.987–3.586), % adherence of 58ST (0.6269–1.006), positive control *S. typhimurium* (0.9680–1.025), *S. typhimurium* + anti-Oma87 (0.7116–0.8150). (E) Survival of HeLa cells following infection with *A. baumannii* strains. The HeLa cells exposed to *A. baumannii* ATCC 19606 survived at the rate of 16.50 ± 3.96%, whereas treatment with anti-Omp34 serum increased survival to 35.94 ± 5.31% (*p* < 0.01). Similarly, HeLa cells infected with *A. baumannii* 58ST exhibited a survival rate of 21.24 ± 1.77%, which improved to 42.90 ± 5.65% (*p* < 0.001) following infection with the bacteria pretreated with anti-Omp34 serum. The lower and upper CIs are negative control (95.48–104.5), positive control 19606 (6.671–26.34), anti-Omp34 + 19606 (22.75–49.13), positive control 58ST (16.85–25.64), and anti-Omp34 + 58ST (28.87–56.93). All columns contained cells cultured with serum, bacteria, or both. Normal HeLa cells were used as the negative control, while HeLa cells infected with *A. baumannii* strains 19606 or 58ST served as the positive controls. (F) Proportion of *A. baumannii* strains internalized in cytochalasin-treated HeLa cells. Values of 5.43 ± 0.72% and 7.23 ± 0.82% internalizations were recorded upon treatment of HeLa cells with cytochalasin D by *A. baumannii* 19606 and 58ST strains, respectively. This contributes to the inhibition of the internalization of *A. baumannii* 19606 and 58ST strains by 94.57 ± 0.72% and 92.77 ± 0.82%, respectively. The lower and upper CIs are control (100), *A. baumannii* 19606 (3.647–7.213), and *A. baumannii* 58ST (5.204–9.262).

The findings from the study revealed a remarkable increase in Ab production within the mice groups. Notably, the group administered with Omp34 displayed a substantially elevated Ab titer in comparison to the control mouse group, as vividly illustrated in Figure 2A. The outcomes of the serum resistance assays are presented in Figure 2B. It was observed that the serum exerted a pronounced significance (*p*  < 0.0001) bactericidal effect on both strains at dilutions of 1:250, 1:500, and 1:1000. However, upon examining the live bacterial populations across two serum dilutions, it was found that *A. baumannii* 19606 displayed a marked statistical significance of *p*  < 0.0001 drop in enhancement in efficacy when transitioning from one dilution to a higher dilution. This is while the bactericidal property of the antisera remained statistically significant (*p*  < 0.0001) even at the highest dilution (1:1000) used. In contrast, *A. baumannii* 58ST exhibited significant susceptibility to the serum at all the applied dilutions. However, while the impact was evident across all dilutions, the statistical significance varied, with no significant difference observed or lower levels of significance (*p*  < 0.05) in the shift from dilution 1:500 to 1:1000 (Figure 2B).

### 3.3. Impact of Immunized Serum on Inhibiting Biofilm Formation

Both the *A. baumannii* strains displayed the capacity to produce biofilms. Notably, the clinical variant called 58ST demonstrated a greater ability to form biofilms, which was expected due to its origin from clinical sources. The existence of Omp34 serum had a preventative impact on the biofilm formation process. A clear distinction with a statistical significance of *p*  < 0.01 was aparent between the control and experimental samples. The positive control samples showed biofilm formation as expected. Nevertheless, anti-Omp34 serum decreased (*p*  < 0.01) biofilm formation by both strains. This impact is clearly visible from the comparative data illustrated in Figure 2C.

### 3.4. *A. baumannii* Adherence, Internalization, and Proliferation in HeLa Cells

Initially, parameters such as the inoculum size, infection periods, washing protocol, and the incorporation of a positive control were optimized for the adherence assay. Through these initial experiments, it was discerned that when employing inoculum densities as low as ≤10^7^ CFU, the number of infected cells for the bacterial strains being evaluated remained relatively low. Consequently, for the subsequent definitive experiments, a density equivalent to around 10^8^ CFU was chosen. This range spanned from 5 × 10^7^ to 5 × 10^8^. Regarding the determination of the most suitable infection time frame, a comprehensive comparison was conducted among durations of 30, 60, 90, 120, and 240 min for the targeted strains. The outcomes illuminated that the peak adherence value was observed at the 240-min mark. Consequently, 240 min was identified as the definitive infection period. During the entire 240-min incubation time frame, it is noteworthy that the monolayers remained intact and healthy. Additionally, the morphology of the infected cells exhibited no noticeable alterations in comparison to the uninfected cells, suggesting the preservation of cellular integrity and function. In the absence of serum, both *A. baumannii* strains tended to enter the host cell. However, anti-Omp34 serum led to a distinct reduction in bacterial internalization. Anti-Omp34 effectively impeded both the adherence and subsequent proliferation of *A. baumannii* 19606, showing significant inhibition (*p*  < 0.0001). *S. enterica* serovar Typhimurium exhibited noteworthy decreased bacterial proliferation in the host cell. Calculation of the percent bacterial adherence, achieved by dividing the overall number of attached bacteria at 4 h postinfection by the total inoculum [28], unveiled no significant disparity between the two strains. This difference resulted in an adherence of 32.87 ± 1.21% for *A. baumannii* ATCC 19606 and 8.17 ± 0.76% for the 58ST strain, as depicted in Figure 2D.

### 3.5. Viability of HeLa Cell Line Following Bacterial Infection

The MTT assay is a useful technique for assessing cell viability by quantifying color intensity, which reflects cellular metabolic activity. In this assay, wells with negative control samples exhibited the deepest purple color, followed by those containing serum. Absorbance measurements at 450 nm further confirmed that the presence of serum significantly enhanced cell viability in the presence of bacteria. This intriguing pattern is visually represented in Figure 2E. When evaluating the viability of HeLa cells, a 24-h period after infection was selected to assess the survival rate. The survival rate of HeLa cells infected with *A. baumannii* ATCC 19606 was 16.50 ± 3.96%, which increased to 35.94 ± 5.31% upon infection with anti-Omp34 treated *A. baumannii* (*p*  < 0.01). HeLa cells infected with the strain 58ST survived 21.24 ± 1.77% (*p*  < 0.0001). However, exposure to bacteria treated with anti-Omp34 serum led to a significantly increased survival rate of 42.90 ± 5.65% (*p*  < 0.001).

### 3.6. Cytochalasin D Impact on HeLa Cell Uptake of *A. baumannii*

Pretreatment of HeLa cells with cytochalasin D yielded significant results. This intervention brought about a substantial decrease in the intracellular bacterial population. Specifically, upon applying cytochalasin D to HeLa cells, an internalization rates of 5.43 ± 0.72% and 7.23 ± 0.82% were recorded for *A. baumannii* 19606 and 58ST strains, respectively. The inhibition of bacterial internalization was reached by 94.57 ± 0.72% and 92.77 ± 0.82% for *A. baumannii* 19606 and 58ST strains, respectively. Internalization by the clinical strain was significantly higher than that of the lab strain (*p*  < 0.05) (Figure 2F).

### 3.7. Microscopic Observations of *A. baumannii* Interactions With HeLa Cells

**Figure 3:**
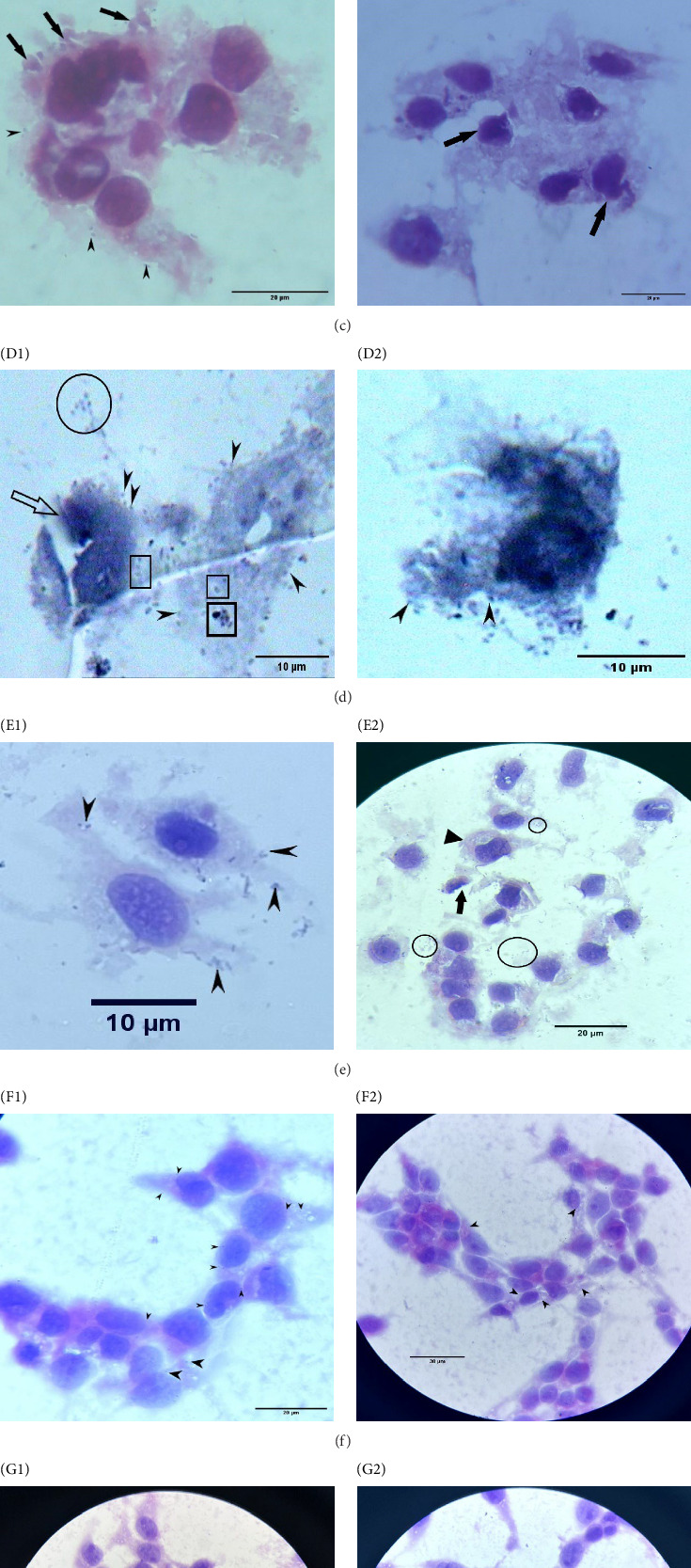
Microscopic images illustrating the internalization of *A. baumannii* within HeLa cells. HeLa CCL-2 cells were infected with *A. baumannii* ATCC 19606 and *A. baumannii* 58ST at a multiplicity of infection (MOI) of 100 for 24 h. Microscopic analysis revealed the interactions between *A. baumannii* and HeLa cells as follows: (A1, A2) normal HeLa cells (A). (B1, B2) *A. baumannii* ATCC 19606 seen vacuolized (arrowheads) (B). The cell membrane blebbing leading to disruption of cytoplasm is evident. Bacterial cells adhering to the inert surfaces are seen as marked with circles. Surface-adherent bacteria are marked with arrow in (B2). (C1, C2) Plenty of anti-Omp34 serum-treated *A. baumannii* ATCC 19606 on the HeLa cell surface or vacuolized (dark arrowheads, C1) (C). Cell blebbing, an indicator of apoptosis (dark arrows), and apoptotic bodies (dark arrows, C2) are noted (C). Apparent HeLa cell blebbing, a characteristic of apoptosis, is seen at the bottom of (C1). (D1, D2) Heavy accumulation of *A. baumannii* 58ST anchored to the HeLa cell (arrowheads) and inert surfaces (circles in D1) and in vacuoles (boxes, D1) (D). The apoptotic cell is marked. HeLa cell necrosis is seen (arrow, D1). HeLa cells undergo extensive degenerative changes. (E1, E2) Anti-Omp34 serum-treated *A. baumannii* 58ST formed clusters in localized areas on the cell cytoplasm or within vacuoles (dark arrowheads) and inert surfaces (circles) (E). HeLa cells retain their healthy structure with minimal response to the pretreated *A. baumannii* 58ST. Apoptotic bodies (arrow, E2) and HeLa cells undergoing apoptosis (triangle, E2) are seen. (F1, F2) HeLa cells occasionally internalize heat-killed bacteria without inducing harm to the host (arrowheads) (F). (G1, G2) Bacterial cells exposed to UV radiation are enclosed within double-membrane-bound organelles (arrowheads) (G), suggesting autophagy. The HeLa cells look healthy. (H1, H2) Formalin-inactivated *A. baumannii* were encapsulated within cytoplasmic vacuoles of HeLa cells (arrowheads, H1) (H). Host cells exhibit cytoplasmic retraction or shrinkage. HeLa cell apoptotic debris is seen in places. Some formalin-killed *A. baumannii* cells are seen adjacent to HeLa cells triggering a reaction of the host cells in the form of membrane protrusions (circles, H2) (H).

HeLa CCL-2 cells underwent infection with the two strains of *A. baumannii*, at an MOI of 100 for a duration of 24 h. The subsequent microscopic analysis provided a detailed observations on the *A. baumannii* and HeLa cells interactions:

The normal HeLa cells are depicted in Figure 3A (A1,A2). Figure 3B (B1,B2) shows *A. baumannii* ATCC 19606 undergoing vacuolization by the host cell (marked with arrowheads). The HeLa cell membrane displays blebbing, which ultimately leads to the disruption of cytoplasm. Bacterial cells are seen adhering to inert surfaces, denoted by circles. The bacterial cells adhering to the cell surface are marked by an arrow in Figure 3B (B2). HeLa cells were heavily populated with *A. baumannii* strain ATCC 19606 that was pretreated with anti-Omp34 serum. The bacteria are seen either on or within the HeLa cell in vacuoles (dark arrowheads, Figure 3C (C1)). The presence of cell blebbing, indicating apoptosis, is notable (dark arrows in Figure 3C (C2)), along with the occurrence of apoptotic bodies (also dark arrows, Figure 3C (C2)). Apparent HeLa cell blebbing, a characteristic feature of apoptosis, is observed at the bottom of Figure 3C (C1). A substantial accumulation of *A. baumannii* 58ST is seen anchored to both HeLa cells (arrowheads) and inert surfaces (circles in Figure 3D (D1)). The bacteria are also observed within vacuoles (boxes in Figure 3D (D1)). The apoptotic cell is marked, while HeLa cell necrosis is evident (arrow in Figure 3D (D1)). The HeLa cells undergo extensive degenerative changes. *A. baumannii* 58ST, which had undergone pretreatment with anti-Omp34 serum, was observed forming clusters in localized regions on the cell cytoplasm, within vacuoles (dark arrowheads), and on inert surfaces (circles). The HeLa cells maintain their healthy structure with minimal response to the pretreated *A. baumannii* 58ST. The presence of apoptotic bodies (arrow in Figure 3E (E2)) and HeLa cells undergoing apoptosis (triangle in Figure 3E (E2)) is noted. HeLa cells were sporadically seen engulfing heat-killed bacteria with no harm to HeLa cells (arrowheads in Figure 3F). Bacterial cells subjected to UV radiation were observed entrapped within membrane-bound organelles (Figure 3G, arrowheads) that indicate autophagy. HeLa cells are healthy. *A. baumannii* cells killed with formalin were seen within cytoplasmic vacuoles (arrowheads, Figure 3H (H1)). The host cells display cytoplasmic retraction or shrinkage, with apoptotic debris visible in certain areas. Formalin-killed bacterial cells were seen positioned next to HeLa cells, instigating the host cells response by membrane protrusions (circles, Figure 3H (H2)).

## 4. Discussion

In order for a bacterium to acquire pathogenicity, it is essential to initiate contact with a specific surface within the host through adherence, preceding the expression of virulence factors that facilitate colonization [30]. Throughout this progression, the bacterium shifts from a planktonic, freely swimming mode of growth to one that is oriented toward a surface. The noteworthy capacity of *A. baumannii* to adhere to and colonize both living (biotic) and nonliving (abiotic) surfaces is acknowledged as a substantial contributing factor to the growing prevalence of nosocomial *A. baumannii* infections. Unlike other pathogens, *A. baumannii* employs a unique strategy known as “persist and resist,” which differs from the conventional toxin expression observed in many other pathogens. The ability of *A. baumannii* to persist and resist, coupled with its remarkable capacity to survive in typically inhospitable conditions, enhances its formidable nature as a pathogen. Transcriptome analysis, comparing mRNA expression profiles of biofilm and planktonic cells, has uncovered a downregulation of genes linked to motility and an upregulation of genes associated with biofilm formation. These genes play a pivotal role in attachment and adherence, crucial steps in establishing a mature biofilm [31]. However, it is noteworthy that reports are challenging this assertion, with clinically isolated *A. baumannii* strains reported to lack the ability to adhere to A549 cells in in vitro studies [24]. Contrary to the preceding report, we found both standard and clinical strains of *A. baumannii* to adhere to and internalize in A549 cells [32]. These conflicting findings underscore the importance of establishing a consistent model for studying the virulence and pathogenicity of *A. baumannii* [16]. Various factors are pivotal for the successful transmission and infection caused by *A. baumannii*, encompassing membrane proteins, cell surface adaptations, antibiotic tolerance, mechanisms of nutrient acquisition, and interactions within bacterial communities [17]. During the current investigation, the acute toxicity of Omp34 was assessed using an animal model. The results indicated mild infiltration of mononuclear leukocytes in the alveolar walls of the lungs and mild, nonspecific inflammation in the spleen tissue. The remaining internal organs displayed a normal structure. Understanding the role of these cell surface structures and antibiotic resistance mechanisms and studying biofilm formation are crucial for the development of effective vaccines against *A. baumannii*. Targeting these factors may help mitigate the antimicrobial resistance or pathogenicity of the bacterium, thereby providing improved immune protection and therapeutic strategies. In this study, both strains of *A. baumannii* showed the capability of biofilm formation. Notably, the clinical isolate 58ST displayed an elevated capacity for forming biofilms, likely due to its origin in a clinical setting. While bacterial biofilm formation was observed in the control samples, the exposure to anti-Omp34 brought about profound extinction in biofilm formation of the bacterial strains. These observations are in parallel to the antibacterial properties of anti-Omp34 serum on *A. baumannii* strains that exerted higher killing property on the clinical strain than the lab strain. The immunized sera exhibited highly noteworthy suppression of biofilm formation in *A. baumannii* 58ST across all dilutions, except for serum dilutions of 1:250 and 1:500, which produced equal effects. The formation of biofilms by *A. baumannii* holds significant implications for its pathogenicity and resistance to antimicrobial agents. Unraveling the sequential processes and regulatory mechanisms underlying formation of biofilm offers invaluable insights for innovative therapeutic approaches. By targeting pivotal factors involved in the creation of biofilms, including mechanisms of adherence, components of the matrix, and pathways of signaling, we can elevate the effectiveness of treatment strategies and surmount the challenges posed by *A. baumannii* infections. Emphasizing research and innovation in this domain is of utmost importance to effectively counter the growing menace of *A. baumannii* and to enhance the quality of patient care within healthcare environments [33]. Gaddy and Actis [34] explored the mechanisms of biofilm formation in *A. baumannii*, which are closely linked to its adherence properties. Genes associated with biofilm formation may also play a role in the coregulation of antimicrobial resistance. Key genes involved in this process include those governing the quorum-sensing system AbaI/AbaR, AbOmpA (OmpA protein), biofilm-associated protein (Bap), the two-component regulatory system BfmRS, PER-1 β-lactamase, EpsA, and PTK. Current experimental therapeutic strategies against *A. baumannii* infections encompass vaccine development, quorum-sensing disruption, nanoparticles, metal ions, natural products, antimicrobial peptides, and phage therapy. A deeper understanding of the interplay between biofilm formation and antimicrobial resistance could aid in identifying novel therapeutic targets, while combined approaches may provide synergistic effects for more effective and safer treatment options [35].

Recent findings have presented new evidence indicating that numerous isolates of *A. baumannii* possess the capability to thrive and propagate within eukaryotic cells, whether they are professional or nonprofessional phagocytes, within a living organism. Smani et al. carried out an experiment involving the knockdown of *omp*33, resulting in a reduction in the attachment and invasion to pulmonary epithelial cells by *A. baumannii*. Consequently, this led to a reduction in *A. baumannii*-induced cytotoxicity. The concentration of Omp34 needed to achieve an IC_50_ was around 10 μg/mL. A more detailed study of cells treated with Omp34 at 10 μg/mL concentration for a duration of 24 h brought to light noticeable changes in morphology. These changes included existence of pseudopodia and vacuoles formation [10]. Our findings support this phenomenon both in live and killed bacterial challenges of HeLa cells. In the infections of HeLa cells by both bacterial strains, the recorded rates of HeLa cell survival were 16.50 ± 3.96% and 21.24 ± 1.77%, respectively. This finding is supported by the lower adherence rate of the clinical strain to HeLa cells that resulted in higher survival rate of HeLa cells compared to that of the lab strain. *A. baumannii* strains 19606 and 58ST adhered HeLa cells at 32.87 ± 1.21% and 8.17 ± 0.76% rates, respectively. Remarkably, the administration of Abs targeting Omp34 caused a significant increase in the survival rates of infected HeLa cells. Specifically, the survival rates saw an augmentation of 2.18 times and 2.01 times, respectively.

*S. enterica* serovar Typhimurium, the positive control, displayed a decrease in intracellular proliferation when exposed to ant-Omp34 sera which was not statistically significant compared to the untreated *S. enterica*. The presence of anti-Omp34 sera did not impact the behavior of this bacterium, likely due to its exclusive presence in *A. baumannii*. This corresponds with the findings reported by Islam et al. [14], who observed that Omp33−36 did not exhibit cross-reaction with sera from patients infected with pathogens other than *A. baumannii*. These findings strongly support the hypothesis that Omp34 plays a significant role in invading host cells. Omp33−36 can promote apoptosis and regulate autophagy in human cells [9, 19]. Wang et al. [9] have put forth evidence suggesting that *A. baumannii* can initiate complete autophagy in HeLa cells, a finding that contrasts with our own results. Our observation of the proliferation of *A. baumannii* within the HeLa cell indicates the incomplete autophagy which is in line with a previous report that proposed Omp33−36 to be able to induce incomplete autophagy, facilitating the replication of *A. baumannii*. This inconsistency could potentially arise from the divergence in the strains of *A. baumannii* employed in our respective studies. This underscores the possibility that different strains of *A. baumannii* might manifest distinct physiological behaviors. Furthermore, it is worth noting that Wang's study subjected HeLa cells to *A. baumannii* for a relatively brief 3-h duration without extending the period of observation. Additionally, their experiments did not yield statistically significant differences in the degradation of p62 [36]. As such, their assertion regarding *A. baumannii* triggering complete autophagy in HeLa cells could be seen as somewhat speculative. This discrepancy warrants further investigation in subsequent research endeavors. The upregulation of autophagy proteins could potentially provide fuel for unregulated bacterial proliferation, potentially setting off heightened inflammatory responses. Our results suggest that *A. baumannii* Omp34 interferes with the fusion process between autophagosomes and lysosomes, hampering the progress of autophagic activity and culminating in incomplete autophagy. In the research conducted by Choi et al., it was observed that preincubation of *A. baumannii* 05KA103 with recombinant AbOmpA led to reduced adherence to and invasion of the epithelial cells. Once inside host cells, *A. baumannii* translocates toward nucleus triggering host cell apoptosis by degradation of DNA [37] as observed in our microscopic examinations. Another mechanism employed by *A. baumannii* to induce apoptosis in host cells involves OmpA, also known as Omp38. This component targets mitochondria, leading to the release of cytochrome c and other proapoptotic molecules that induce apoptosis [38]. Recent studies suggest that strains of the *Acinetobacter calcoaceticus*–*A. baumannii* complex can induce the formation of autophagic vacuoles when associated with apoptosis in human epithelial cells [39]. Understanding the interplay between apoptosis and autophagy induced by these strains could yield crucial insights into *Acinetobacter* infections' pathogenicity and identify potential therapeutic targets. Suppressing autophagy allows the bacterium to flourish within host cells, reproducing within autophagosomes and ultimately leading to cytotoxicity. Our research demonstrates considerable damage to HeLa cells following viable *A. baumannii* infection, emphasizing the hindrance of the autophagic process by the infecting bacteria. To investigate the role of microfilaments and microtubules in *A. baumannii* cell invasion, we utilized cytoskeleton inhibitors, specifically cytochalasin D, on epithelial cells. Treating these cells with cytochalasin D significantly inhibited *A. baumannii* cell invasion. The reduction in intracellular bacteria after cytochalasin D treatment confirms the role of actin-mediated processes in *A. baumannii* uptake by epithelial cells, which suggest the vital role of actin filaments in facilitating bacterial invasion. The importance of microtubules in the invasion process also implies their contribution to *A. baumannii*'s internalization mechanisms. Our findings provide strong evidence that *A. baumannii* exploits both microfilament and microtubule-dependent mechanisms to invade epithelial cells, providing insights into the intricate interactions between the bacterium and host cells during infection. Notably, the counts of intracellular bacteria reduced by 94.57 ± 0.72% and 92.77 ± 0.82% in the strains ATCC 19606 and 58ST, respectively. Additional confirmation tests were undertaken by utilizing inactivated *A. baumannii*, which were then introduced to HeLa cells. These tests were carried out to determine whether HeLa cell invasion involves mechanisms that rely on microfilaments and microtubules. Different interactions between HeLa cells and UV-treated or formalin-fixed *A. baumannii* were observed in the present study. HeLa cells entrapped UV-treated bacteria within double-membrane vacuoles, indicative of autophagy, while *A. baumannii* killed with formalin were enclosed by HeLa cells in the vacuoles, both singly and in clusters. Autophagy plays a significant role in the innate immune response against bacterial infections [40, 41]. It has been illustrated that exposure of A549 cells to live *A. baumannii* for 2 h led to a noteworthy 50% increase in the lysosome count. Conversely, when HeLa cells were challenged with heat-killed *A. baumannii*, no substantial rise was noted in lysosomes. This implies that viable *A. baumannii* can trigger lysosome formation within host cells, a capability absent in heat-killed *A. baumannii* [42]. This firmly suggests that *A. baumannii* employs microfilament and microtubule-dependent mechanisms to invade epithelial cells. The invasion of epithelial cells by *A. baumannii* exhibits variability depending on bacterial strains and cell types. Cellular invasion is facilitated through mechanisms involving both microfilaments and microtubules. The microbial component Omp34 plays a crucial role in mediating the adherence of *A. baumannii* to and invasion of epithelial cells. The insights gained from this research offer a valuable understanding of the initial stages of *A. baumannii* pathogenesis during bacterial infections. Overall, our results present novel perspectives into *A. baumannii* pathogenesis. By unraveling the intricate interactions between the bacterium and host cells, we enhance our understanding of how *A. baumannii* eludes the immune system and triggers disease. This understanding is pivotal for devising targeted interventions to effectively combat *A. baumannii* infections. Our research aimed to investigate the distinct characteristics of *A. baumannii* strains with varying susceptibility to anti-Omp34 Abs and their invasion of human cervical carcinoma epithelial cells (HeLa cells). Therefore, we focused on multiple aspects of *A. baumannii* interactions with HeLa cells, including cell viability, adherence, serum resistance, internalization, and intracellular proliferation, both in the presence and absence of anti-Omp34 sera. However, this focus, along with the limited number of strains analyzed, may represent a limitation of the study.

Our findings significantly enhance the understanding of *A. baumannii* pathogenicity, particularly regarding its adherence, invasion, and immune evasion mechanisms. Prior studies have highlighted the necessity of bacterial adherence to host surfaces as a crucial first step for pathogenesis, facilitating colonization and subsequent infection. While it has been established that *A. baumannii* demonstrates a strong ability to adhere to both biotic and abiotic surfaces, our research provides novel insights into the role of Omp34 in modulating these interactions. Our study supports the growing body of evidence indicating that *A. baumannii* possesses the ability to survive and proliferate within eukaryotic cells, similar to intracellular bacterial pathogens. Previous work has demonstrated that the deletion of omp33 reduces *A. baumannii* adherence and invasion of pulmonary epithelial cells, leading to decreased cytotoxic effects. We extend these findings by demonstrating that Omp34-targeting Abs significantly enhance HeLa cell survival by limiting bacterial internalization and damage. This suggests that Omp34 plays a critical role in mediating host–pathogen interactions and may serve as a potential target for therapeutic intervention. Additionally, our findings shed new light on the interplay between *A. baumannii* infection and host cell autophagy. Previous studies suggested that *A. baumannii* might trigger complete autophagy, aiding in bacterial clearance. However, our study suggests that Omp34 interferes with autophagic processes, resulting in incomplete autophagy that facilitates bacterial survival and proliferation within host cells. Moreover, we provide strong evidence that *A. baumannii* exploits both microfilament- and microtubule-dependent mechanisms for host cell invasion.

In conclusion, our study advances the field by offering novel perspectives on *A. baumannii*'s intracellular survival strategies, immune evasion mechanisms, and potential therapeutic targets. The identification of Omp34 as a key factor in adherence, invasion, and biofilm formation provides a foundation for future vaccine development.

## Data Availability

Data sharing is not applicable to this article as no new data were created or analyzed in this study.
